# Acute kidney injury in the era of big data: the 15^th^ Consensus Conference of the Acute Dialysis Quality Initiative (ADQI)

**DOI:** 10.1186/s40697-016-0103-z

**Published:** 2016-02-26

**Authors:** Sean M. Bagshaw, Stuart L. Goldstein, Claudio Ronco, John A. Kellum, for the ADQI 15 Consensus Group

**Affiliations:** Division of Critical Care Medicine, Faculty of Medicine and Dentistry, University of Alberta, 2-124E, Clinical Sciences Building, 8440-112 ST NW, Edmonton, T6G 2B7 Canada; Center for Acute Care Nephrology, Cincinnati Children’s Hospital Medical Center, Cincinnati, OH USA; Department of Nephrology, Dialysis and Transplantation, International Renal Research Institute of Vicenza, San Bortolo Hospital, Vicenza, Italy; Center for Critical Care Nephrology, Department of Critical Care Medicine, University of Pittsburgh, Pittsburgh, PA USA

## Abstract

The world is immersed in “*big data*”. Big data has brought about radical innovations in the methods used to capture, transfer, store and analyze the vast quantities of data generated every minute of every day. At the same time; however, it has also become far easier and relatively inexpensive to do so. Rapidly transforming, integrating and applying this large volume and variety of data are what underlie the future of big data. The application of big data and predictive analytics in healthcare holds great promise to drive innovation, reduce cost and improve patient outcomes, health services operations and value. Acute kidney injury (AKI) may be an ideal syndrome from which various dimensions and applications built within the context of big data may influence the structure of services delivery, care processes and outcomes for patients. The use of innovative forms of “information technology” was originally identified by the Acute Dialysis Quality Initiative (ADQI) in 2002 as a core concept in need of attention to improve the care and outcomes for patients with AKI. For this 15^th^ ADQI consensus meeting held on September 6–8, 2015 in Banff, Canada, five topics focused on AKI and acute renal replacement therapy were developed where extensive applications for use of big data were recognized and/or foreseen. In this series of articles in the *Canadian Journal of Kidney Health and Disease*, we describe the output from these discussions.

## ABRÉGÉ

Le monde nage actuellement dans une mer de données informatiques. L’apparition de mégadonnées a entraîné des changements majeurs dans la façon de saisir, de transférer, de stocker et d’analyser la multitude de données générée chaque minute de chaque jour. Parallèlement, il est aussi plus facile de gérer ces informations et de le faire à un coût relativement moindre. La capacité de transformer, d’intégrer et d’appliquer rapidement la variété et le volume considérable de données est ce sur quoi repose le futur des mégadonnées. Le traitement des mégadonnées ainsi que leur analyse prévisionnelle dans le système de santé se veut très prometteur pour favoriser l’innovation, réduire les coûts, apporter des changements favorables au fonctionnement et à la portée des services ainsi que pour améliorer le pronostic des patients. Il semble que l’insuffisance rénale aiguë (IRA) soit un syndrome idéal à partir duquel les différents aspects et applications mis en place pour gérer les mégadonnées pourraient influencer les modèles existants de prestation de services et d’offre de soins et, par extension, l’évolution de l’état de santé des patients. L’utilisation de formes novatrices pour assimiler les technologies et l’information a d’abord été identifiée en 2002 par l’*Acute Dialysis Quality Initiative (ADQI)* en tant que concept nécessitant une attention particulière et qui pourrait améliorer les soins et le pronostic des patients atteints d’IRA. Lors de la 15e réunion annuelle de concertation de l’ADQI qui s’est tenue du 6 au 8 septembre 2015 à Banff, au Canada, on a mis l’accent sur cinq thèmes reliés à l’IRA et aux thérapies de remplacement rénal. Ils ont été développés dans des cadres où l’on reconnaissait ou prévoyait l’application étendue des mégadonnées. Dans une série d’articles qui paraîtra dans le *Canadian Journal of Kidney Health and Disease*, nous ferons ressortir les conclusions de ces discussions.

## Introduction

*“In God we trust; all others must bring data.”* – W. Edwards Deming

We are immersed in a world characterized by “*Big Data*” [1] The term big data was likely first used in 1997 to describe the mounting challenges for computing systems to manage immense data sets [2]. Indeed, according to Wikipedia, big data is “*a broad term for data sets so large or complex that traditional data processing applications are inadequate*” [3]. This has necessitated radical innovation in the methods used to capture, transfer, store and analyze the utterly incomprehensible volumes of data generated in society every minute of every day. Yet, at the same time, it has become relatively easy and inexpensive to do so. Technological innovations have enabled many of our day-to-day activities to be “data-fied” and leave unique and discrete digital traces, from personal (i.e., web searching, wearing a smart watch) to professional tasks (i.e., use of electronic clinical information systems or medical records). Indeed, nearly all forms of new technology introduced today have integrated computers and sensors. Our capacity to integrate this large volume and variety of data, relatively quickly (three of the four characteristic “V”s of big data: *volume, velocity, variety and veracity*) are what underlies the future and promise of big data. Its potential has brought about a revolution of changes in the fields of science (i.e., genomics, meteorology, astrophysics), business (i.e., retail, banking), and media and communications (i.e., data exhaust, and mouse clicks on Facebook, Amazon, and Google).

## Big data in healthcare

The use of big data and predictive analytics in healthcare also holds great promise to drive innovation, increase efficiency, improve outcomes for patients and reduce costs while advancing the value of service delivery [4]. Big data in healthcare aims to introduce new technologies to acquire, integrate and analyze data to guide clinical research, optimize hospital operations and inform “best” clinical practice [5]. However, “data” is not synonymous with information or for that matter with knowledge. Big data has challenges. The sheer quantity of data is growing faster than our capacity to aptly utilize and apply it. Similarly, it remains uncertain whether unconventional sources and unstructured data, clearly a departure from classical epidemiological teaching, can be appropriately and/or suitably applied to inform about health and disease. Examples include the use of social media to estimate obesity prevalence or Google Flu to trend influenza rates [6, 7]. Chunara et al estimated population obesity rates by performing a cross-sectional study correlating described user interests on the social network Facebook in New York City neighborhoods with national obesity prevalence rates. In this study, a greater proportion of user activity-interests in television correlated with a higher prevalence of obesity [6]. Alternatively, during the 2013 influenza season, Google Flu Trends (GFT) grossly over-estimated the incidence of influenza compared with Centers for Disease Control (CDC) surveillance reports, attributed largely to modifications and imperfections in the GFT search algorithm [7].

The practice of medicine has traditionally relied on the skills of clinicians, which to a great extent relies on “clinical intuition”. In acute care settings, clinicians commonly size up a clinical situation by, often subconsciously, integrating multi-modal sources of data from history, physical examination, diagnostic imaging and laboratory investigations, along with temporal visual cues, to inform their actions. The complexity of big data at the bedside is rapidly growing, and while still modest, the volume (along with velocity, variety and veracity) can greatly exceed that which clinicians can conceivably integrate and consciously analyze into their clinical decision making. Naturally then, experienced clinicians use a variety of heuristics to guide their clinical assessment and bedside decision-making. Part of the appeal of big data is the potential for computers and capacity for complex analytics to reproduce (and improve on) these clinical heuristics to better inform patient care, along with improve healthcare system operations. Finally, issues related to privacy, security and ownership of data have understandably emerged to worry patients, providers and custodians of health data. These challenges aside, big data will certainly continue to influence the nature of healthcare, if not reshape and define it, in terms of prognostics, surveillance, operations and service delivery, across a broad continuum for the foreseeable future.

## How then can big data apply to acute kidney injury (AKI)?

We contend that AKI may be an ideal syndrome to use big data to develop applications to guide and influence the structure of services delivery, care processes and outcomes for patients [8]. The use of innovative forms of “information technology” was originally identified by the Acute Dialysis Quality Initiative (ADQI) in 2002 as a core concept in need of attention to improve the care and outcomes for patients with AKI [9].

Indeed, AKI may be an important syndrome to focus on for a variety of reasons [10]. First, AKI is common in hospitalized patients [11]. Second, AKI imposes a significant increased risk for major morbidity, including chronic kidney disease and accelerated progression to end-stage kidney disease and death [12, 13]. Third, AKI is expensive [14, 15]. Patients suffering an episode of AKI consume greater resources and incur higher costs, largely from intensified monitoring, investigations, and support and longer hospital stays. Finally, and perhaps most importantly, recent data highlight how the care of patients with AKI is suboptimal, even poor, and characterized by numerous deficiencies and systematic failings, implying much may in fact be avoidable [16, 17]. Accordingly, based on these assertions, we believed that an ADQI consensus meeting focused on how big data could transform and/or translate into tangible improvement in AKI care was justified and needed.

## AKI in the era of big data

For this ADQI consensus meeting, we proposed five discrete yet overlapping topics within the broad realm of critical care nephrology, specifically focused on AKI and RRT, whereby we recognized and/or foresaw extensive applications for use of big data. The first topic focused the concept of development and utilization of predictive analytics, forecasting and risk identification applications for AKI, leveraged on the existing and/or planned integration of electronic medical records (EMR) and clinical information systems (CIS) available at the point of care. Conceptually, this would enable a hospital-wide platform for clinical risk prediction and integration decision support to mitigate “avoidable” episodes of AKI [18]. The second topic focused on particular methodology to develop novel applications to detect and classify AKI among hospitalized patients using EMR/CIS platforms. Conceptually, this would enable the detection of AKI at the earliest opportunity and provide maximal lead time to mitigate avoidable propagation of AKI or harm [19–21]. The third topic focused on the concept of automated electronic alerting for patients either at-risk or who have had overt AKI detected. This topic focused on the methods and forms of communicating alerts regarding AKI to inter-disciplinary providers along with the integration, context and format of decision support [22–24]. The fourth topic aimed to revisit how AKI is currently “coded” across administrative databases [25–27]. This topic also focused on how existing administrative, clinical and research database infrastructure may be leveraged for “risk identification” for large scale pragmatic registry-based clinical trials or used for quality assurance, outcome, health system utilization focused projects [28, 29]. The final topic aimed to establish how big data could trace the arc of care for a patient who suffered an episode of AKI associated with a discrete hospitalization by optimally utilizing a wide variety of data sources [30]. Conceptually, this may represent the critical pathway of a patient as they transition through various aspects of a health system that can inform on the “natural” history of AKI.

In this series of articles in the *Canadian Journal of Kidney Health and Disease*, we aim to describe the output from our discussions that took place during the 15^th^ ADQI consensus conference on “Acute Kidney Injury in the Era of Big Data” in Banff, Canada on September 6–8, 2015.

## ADQI methodology

The methodology of ADQI consensus conferences are well developed and have been further refined over the last decade, as previously described [31, 32]. In brief, the ADQI methodology begins with a systematic search and appraisal of scientific evidence to identify emerging priorities in the field. This is followed by a surveillance of current practice, evidence implementation and/or integration of evidenced-based techniques, along with identification of key areas where knowledge or care gaps are prevalent.

For the 15^th^ ADQI consensus conference, the meeting chairs selected the broad theme of “*Acute Kidney Injury in the Era of Big Data*” to acknowledge the evolving nature and growing importance of information and technology in the care of patients with AKI. The meeting chairs invited a diverse expert panel representing relevant disciplines (i.e., nephrology, critical care, pediatrics, pharmacy, epidemiology, biostatistics, and informatics) from a variety of countries and scientific societies around this theme. The methodology utilized a process of both “evidence appraisal” and “expert panel” [33].

The activities of the ADQI consensus conferences have been traditionally partitioned into three discrete phases: pre-conference, conference and post-conference. In the preconference phase, key topics for each work group are developed and refined, work groups are established (4–6 members per group), and specific topics are assigned. Each work group developed a series of key questions focused on their topic, performed a systematic review of the literature, and summarized the current state of knowledge to facilitate further refinement of their key questions from which discussion and consensus statements could be developed.

The conference phase is characterized by a series of breakout sessions where each work group aims to identify key issues, grade the evidence, classify the current state of consensus and develop summary statements and where applicable, provide recommendations. These sessions alternate with plenary sessions, where each work group iteratively presents their questions addressing each identified issue along with draft consensus statements. Throughout this process, each question and statement are discussed, debated, and further refined as necessary. This process also aims to facilitate translation of identified knowledge gaps into future agendas for research. The conference chairs act as facilitators during both the breakout and plenary sessions.

During the post-conference phase, each work group consolidates their findings, specifically their background, rationale and evidence synthesis with their key questions and consensus statements in a concise manuscript. Each manuscript is circulated among work groups for comment and further edited for style and uniformity by the conference chairs.

The broad objectives of ADQI are to provide expert-based statements and interpretation of current knowledge for use by clinicians according to professional judgment and identify evidence care gaps to facilitate research priorities. For the 15^th^ ADQI consensus conference, the focus was on the rapid emergence of big data in healthcare and how it may impact the field of critical care nephrology. We believe that the five papers presented in this series in the *Canadian Journal of Kidney Health and Disease* will provide a broad overview of the current status of big data in AKI and a roadmap for its future applications to improve care delivery and outcomes for patients.
